# Predictive Analytics for Inpatient Postoperative Opioid Use in Patients Undergoing Mastectomy

**DOI:** 10.7759/cureus.23079

**Published:** 2022-03-11

**Authors:** Isabella M Dolendo, Anne M Wallace, Ava Armani, Ruth S Waterman, Engy T Said, Rodney A Gabriel

**Affiliations:** 1 Anesthesiology, University of California San Diego, La Jolla, USA; 2 Surgery, University of California San Diego, La Jolla, USA; 3 Anesthesiology, University of California San Diego, San Diego, USA

**Keywords:** analgesia, acute pain, mastectomy, machine learning, opioids

## Abstract

Introduction: The use of opioids in mastectomy patients is a particular challenge, having to balance the management of acute pain while minimizing risks of continuous opioid use postoperatively. Despite attempts to decrease postmastectomy opioid use, including regional anesthetics, gabapentinoids, topical anesthetics, and nonopioid anesthesia, prolonged opioid use remains clinically significant among these patients. The goal of this study is to identify risk factors and develop machine-learning-based models to predict patients who are at higher risk for postoperative opioid use after mastectomy.

Methods: In this retrospective cohort study, we collected data from patients that underwent mastectomy procedures. The primary outcome of interest was defined as oxycodone milligram equivalents (OME) greater than or equal to the 75% of OME use on a postoperative day 1. Model performance (area under the receiver-operating characteristics curve (AUC)) of various machine learning approaches was calculated via 10-fold cross-validation. Odds ratio (OR) and 95% confidence intervals (CI) were reported.

Results: There were a total of 148 patients that underwent mastectomy and were included. The medium (quartiles) postoperative day 1 opioid use was 5 mg OME (0.25 mg OME). Using multivariable logistic regression, the most protective factors against higher opioid use was being postmenopausal (OR: 0.13, 95% CI: 0.03-0.61, p = 0.009) and cancer diagnosis (OR: 0.19, 95% CI: 0.05-0.73, p = 0.01). The AUC was 0.725 (95% CI: 0.572-0.876). There was no difference in the performance of other machine-learning-based approaches.

Conclusions: The ability to predict patients’ postoperative pain could have a significant impact on preoperative counseling and patient satisfaction.

## Introduction

More than 100,000 mastectomies are performed in the United States each year [1]. Chronic pain is one of the most common complications of breast surgery, estimated to affect 20-30% of patients [2]. Postmastectomy pain syndrome (PMPS) is a type of chronic neuropathic pain disorder that may occur after mastectomies, particularly when tissue is removed from the upper outer quadrant of the breast or axilla, where major nerves are particularly vulnerable to nerve injury [3,4]. The pain is characterized by burning, shooting, stabbing, tingling, or stinging pain and is typically localized to the axilla, anterior/lateral chest wall, and/or medial upper arm [5]. PMPS can be severe enough to cause long-term disabilities, interfering with sleep and performance of daily activities [6]. Retrospective studies found that PMPS can cause persistent stress that can increase an individual’s susceptibility to stress-related physical and mental health problems [6].

Additionally, chronic postsurgical pain is increasingly recognized as a significant contributor to chronic opioid use [7]. With overdoses involving prescription opioids quadrupling since 1999, the United States is facing the challenge of reducing misuse of prescription opioids [8]. Women undergoing simple mastectomy have been previously identified to be at risk for chronic opioid use [9]. Persistent opioid use among patients undergoing breast procedures has been estimated to be as high as 58% [10]. Many current attempts to reduce the use and abuse of opioids focus on the management of chronic pain. The use of opioids in surgical patients is a particular challenge, having to balance the management of acute pain while minimizing risks of continuous opioid use postoperatively [11]. Despite attempts to decrease postmastectomy opioid use, including regional anesthetics, gabapentinoids, topical anesthetics, and nonopioid anesthesia, prolonged opioid use remains clinically significant among these patients [12].

There has been evidence suggesting a close correlation between acute pain and opioid use with that of chronic pain and opioid use [2,13,14]. Therefore, the goal of this study was to identify risk factors and to develop machine-learning-based models to predict patients who are at higher risk for acute postoperative opioid use after mastectomy. The ability to predict patients’ postoperative pain could have a significant impact on preoperative counseling and patient satisfaction. Additionally, the predictive ability may direct different pain management techniques perioperatively (i.e., intercostal cryoanalgesia or two-level paravertebral catheters) [15-17].

This article was previously presented as a meeting abstract at the 2021 International Anesthesia Research Society on May 15-16, 2021 and the 2021 Association of University Anesthesiologists Annual Meeting on May 13-14, 2021.

## Materials and methods

Study population

This retrospective study was approved by the UC San Diego Human Research Protections Program (Intuitional Review Board Protocol#200392) for the collection of patient data from our electronic medical record system and waived the need for informed consent. All patients that underwent elective mastectomy performed by either of the two surgeons (AW and AH) at our institution from 2019 to 2020 were included in the analysis. Data was manually collected from the electronic medical record system by ID and RG. All methods were carried out in accordance with relevant guidelines and regulations. All patients received a paravertebral T4 catheter preoperatively with ropivacaine 0.2% infusing at 8 mL per hour with a 4 mL as-need bolus every 30 minutes (every 60 minutes if the patient had bilateral catheters). Their catheters were removed on postoperative day 2. All patients underwent general anesthesia for surgery. Postoperatively, the analgesic plan included scheduled oral acetaminophen, paravertebral nerve block infusion, and as-needed oxycodone 5-10 mg every 4 hours. Increased frequency or dosing of oxycodone was ordered by the surgical team. The paravertebral catheter was managed by the regional anesthesia team. All patients were discharged from the hospital no earlier than postoperative day 2.

Primary endpoint

The primary outcome of interest (binary) was total opioid use on postoperative day 1 less than or greater than or equal to the third quartile of opioid use. We chose postoperative day 1 opioid use as an outcome measurement for pain as this represented a consistent 24-hour period where data was available and in which the acute pain associated with the surgery may be at its peak. This was calculated by collecting data from all intravenous/oral morphine use, intravenous/oral hydromorphone use, intravenous fentanyl use, oral tramadol use, oral oxycodone use, and oral hydrocodone use during postoperative day 1. These totals were then converted to oral oxycodone milligram equivalents (OME).

Predictor variables

We collected a number of patient and surgical characteristics that would be known preoperatively. The surgical covariates included: involvement of axillary node dissection, breast implant/expander placement, if surgery was bilateral (versus unilateral), and indication of surgery (cancer vs. non-cancer (i.e., prophylactic surgery, transgender surgery)). Patient characteristics included race (White vs. non-White race), sex, body mass index (BMI) (categorized to less than or greater than or equal to 35 kg/m^2^), age, non-English speaker, transgender, postmenopausal, American Society of Anesthesiologists physical status classification score, diabetes mellitus, chronic kidney disease, obstructive sleep apnea, depression, anxiety, attention-deficit hyperactive disorder, active marijuana use, active substance abuse, active smoker, chronic opioid use, fibromyalgia, chronic pain, hypertension, active alcohol use, chronic obstructive pulmonary disease, coronary artery disease, preoperative systolic blood pressure, and preoperative heart rate.

Statistical analysis

All statistical analysis was performed using the R language (Version 6.2.3). To compare patient and surgical characteristics between both cohorts (patients using less than the third quartile of total OME versus greater than or equal to the third quartile of total OME on postoperative day 1), we used student’s t-test and chi-square test to compare continuous and categorical variables, respectively. Next, we built a predictive model using multivariable logistic regression with variable selection. The dependent variable was whether or not the patient used greater than or equal to the third quartile of OME on postoperative day 1. Initially, all covariates were included as independent variables in the initial model. We then used a combination of forward selection and backward elimination based on the Akaike Information Criterion, to develop the final model. Odds ratio (OR) and their associated 95% confidence interval (CI) were reported for each covariate included in this model. Next, we developed predictive models using other machine learning approaches, including 1) multivariable logistic regression using all variables (no variable selection); 2) ridge regression; 3) lasso regression; and 4) elastic net regression. Ridge regression is similar to maximum likelihood estimation, except a shrinkage penalty is applied to each regression coefficient, and thus it shrinks the coefficients toward zero. This uses an L2-penalization. The shrinkage penalty is weighted based on a tuning parameter λ. This provides an advantage in the bias-variance tradeoff, in which increases in λ may lead to decreased variance but increased bias. Ridge regression uses all predictor variables in the final model, whereby no coefficients will be equal to exactly zero. In contrast, while lasso has very similar properties to ridge regression, it allows smaller, more parsimonious models, (i.e., some regression coefficients may equal zero). In contrast to ridge regression, lasso uses an L1-penalization. The optimal λ value was determined via cross-validation and was used for the final models. Elastic net regularized generalized linear models is a method that linearly combines the L1 and L2-penalization from lasso and ridge regression.

To assess the performance of each model, we calculated the area under the receiver-operating characteristics curve (AUC) to evaluate discrimination and the Hosmer-Lemeshow (HL-test) to evaluate goodness-of-fit. We performed 10-fold cross-validation to further assess model performance. The entire dataset was split into 10 equal “folds,” in which one fold served as the validation set and the remaining folds were the training set. The model was trained on the training set and tested on the validation set. The AUC was calculated on the validation set. This was repeated 10 times, whereby each fold had an opportunity to serve as a validation set. The average AUC and 95% CI was calculated thereafter. We used the student’s t-test to calculate the difference in the mean AUC within each machine learning approach as it compared to the reference model (multivariable logistic regression with variable selection).

## Results

There were a total of 148 patients that underwent a mastectomy and were included in our final analysis. The medium (quartiles) postoperative day 1 opioid use was 5 mg OME (0.25 mg OME) with a range from 0 mg to 211.2 mg OME. We separated the population into two cohorts, one with less than the third quartile of OME (25 mg OME) and another with greater than or equal to the third quartile (n = 38). Table 1 lists the patient characteristics in both cohorts. On crude analysis, the only covariate that was statistically significantly different in both cohorts was whether the patient was postmenopausal (42.7% versus 21.1% in the lower versus higher opioid use cohorts, respectively, p = 0.03).

**Table 1:** Patient characteristics of the two study cohorts. ADHD: attention-deficit hyperactive disorder; OME: oxycodone milligram equivalents; SD: standard deviation; ASA: the American Society of Anesthesiologists; COPD: chronic obstructive pulmonary disease

		OME <75% quartile		OME ≥75% quartile		
		n	%	n	%	p-value
Total		110		38		
Mastectomy surgery						
	Node dissection involvement	40	36.4	12	31.6	0.74
	Tissue expander placement	21	19.1	9	23.7	0.71
Bilateral surgery		58	52.7	22	57.9	0.72
Cancer diagnosis		83	75.5	23	60.5	0.12
Age (years), mean (SD)		45.5 (17.1)		40.5 (13.1)		0.06
Male sex		11	10.0	3	7.9	0.95
BMI ≥ 35 kg/m^2^		11	10.0	2	5.3	0.58
White race		61	55.5	26	68.4	0.23
Non-English speaker		21	19.1	4	10.5	0.34
Transgender		24	21.8	11	28.9	0.51
Postmenopausal		47	42.7	8	21.1	0.03
ASA physical status score						0.25
	1	13	11.8	7	18.4	
	2	46	41.8	19	50.0	
	3	51	46.4	12	31.6	
Active smoker		1	0.9	2	5.3	0.33
Active alcohol use		43	39.1	19	50.0	0.32
Chronic opioid use		0	0.0	2	5.3	0.11
Illicit drug use		1	0.9	1	2.6	0.99
Marijuana use		6	5.5	5	13.2	0.23
Preoperative vital signs						
	Systolic blood pressure	116.5 (17.3)		112.8 (14.1)		0.19
	Heart rate	77.1 (14.2)		73.5 (11.6)		0.13
Comorbidities						
	Diabetes mellitus	6	5.5	1	2.6	0.79
	Chronic kidney disease	4	3.6	0	0.0	0.54
	Obstructive sleep apnea	5	4.5	3	7.9	0.71
	Depression	30	27.3	6	15.8	0.23
	Anxiety	24	21.8	9	23.7	0.99
	ADHD	3	2.7	3	7.9	0.36
	Fibromyalgia	2	1.8	2	5.3	0.58
	Hypertension	29	26.4	4	10.5	0.07
	COPD	1	0.9	1	2.6	0.99
	Asthma	16	14.5	2	5.3	0.22
	Congestive heart failure	0	0.0	0	0.0	0.99
	Coronary artery disease	1	0.9	0	0.0	0.99

**Figure 1:**
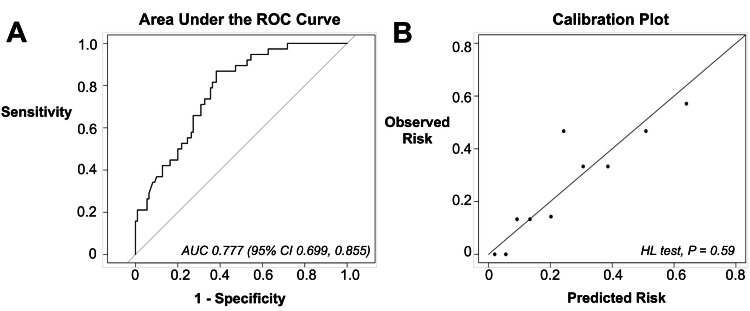
Performance of the multivariable logistic regression with variable selection predicting patients at risk for higher acute opioid use on postoperative day 1: A) area under the receiver operating characteristics curve and B) calibration plot illustration goodness-of-fit. AUC: area under the receiver-operating characteristics curve; CI: confidence interval; HL: Hosmer–Lemeshow; ROC: receiver-operating characteristics

We performed a multivariable logistic regression model with variable selection in order to identify specific covariates associated with opioid use and to develop a predictive model. From this model (Table 2), the most protective factors against higher opioid use were being postmenopausal (OR: 0.13, 95% CI: 0.03-0.61, p = 0.009) and cancer diagnosis (OR: 0.19, 95% CI: 0.05-0.73, p = 0.01). The predictive model had an AUC of 0.777 (95% CI: 0.699-0.855) and the HL-test demonstrated adequate goodness-of-fit (p = 0.59) (Figure 1). On 10-fold cross-validation, the average AUC was 0.725 (95% CI: 0.572-0.876).

**Table 2:** Results of the multivariable logistic regression, in which the outcome was oxycodone equivalents ≥ 75% quartile on postoperative day 1. The final model was developed by a combination of forward selection and backward elimination based on the Akaike Information Criterion. Only covariates with p<0.2 were allowed to stay in the final model. CI: confidence interval; OR: odds ratio

		OR (95% CI)	p-value
Postmenopausal		0.13 (0.03-0.61)	0.009
Age (years)		1.04 (0.99-1.09)	0.12
Mastectomy with tissue expander placement		2.12 (0.73-6.17)	0.17
Bilateral surgery		0.36 (0.11-1.17)	0.09
Cancer diagnosis		0.19 (0.05-0.73)	0.01
White race		2.95 (1.17-7.42)	0.02
Depression		0.31 (0.09-1.07)	0.06
Substance abuse history		16.11 (0.66-391.8)	0.09
Active smoker		30.99 (1.36-703.6)	0.03
Hypertension		0.28 (0.06-1.20)	0.09
Asthma		0.19 (0.03-1.09)	0.06

We compared the performance of our multivariable logistic regression model with variable selection to other model types, including: 1) multivariable logistic regression including all variables; 2) ridge regression; 3) lasso regression; and 4) elastic net regression. On 10-fold cross-validation, the average AUC for multivariable logistic regression including all variables was 0.763 (95% CI: 0.646-0.880); for ridge regression was 0.775 (95% CI: 0.562-0.988), for lasso was 0.799 (95% CI: 0.645-0.953), and elastic net regression was 0.801 (95% CI: 0.647-0.955). Compared to our reference model, there were no statistically significant differences between AUCs among each model: multivariable logistic regression including all variables (p = 0.92), ridge regression (p = 0.39), lasso (p = 0.22), and elastic net regression (p = 0.15).

## Discussion

We developed a predictive model to identify patients who are at high risk for higher acute opioid use after mastectomy. Using different machine learning approaches, we found that logistic regression performed just as well as other methodologies. The model had excellent discrimination and included predictors such as postmenopausal, age, race, bilateral surgery, mastectomy with tissue expander placement, cancer diagnosis, depression, substance abuse history, smoking, hypertension, and asthma.

We showed that being postmenopausal was a protective factor against higher opioid use. The literature suggests that the association between menopause and changes in pain sensitivity remains unclear. One study showed a longer cold withdrawal time among post-menopausal women in a cold pressor test [18]. Their performance in cold pressor tests suggests a higher pain tolerance among this group, which could help explain this population’s decreased opioid use. The study also found no effect of the menstrual cycle on cold withdrawal time, suggesting the difference in postmenopausal women cannot be solely explained by varying hormone levels [18]. Another study that measured brain mu-opioid receptor binding by positron emission tomography (PET) found that mu-opioid binding declined in postmenopausal women, predicting lower sensitivity to mu-opioid analgesics [19]. This may suggest that the decrease in opioid use that we observed may not be related to mu-opioid receptor differences and could be explained by other elements such as psychological factors. These studies in addition to our findings indicate a need for additional research to explore post-menopause as a protective factor against increased opioid use and to elucidate the mechanisms of this effect.

A cancer diagnosis was found to be another protective factor. The experience of a cancer diagnosis and subsequent treatment poses unique physical, emotional, and mental challenges. Studies have demonstrated that the majority of cancer patients exhibit resilience when faced with highly disruptive events and that patients who approach surgery with resilience and optimistic attitudes are less likely to have high intensity acute postoperative and chronic pain [20-23]. The non-cancer group included patients who underwent prophylactic and gender-affirming mastectomies. When women who are at high risk of developing breast cancer are deciding whether to proceed with prophylactic mastectomies versus breast-conserving measures, fear and anxiety appear to be the main influence for decision-making [24]. A study found that women who elect to undergo a contralateral prophylactic mastectomy have greater anxiety and overall fear of breast cancer recurrence compared with those who chose unilateral mastectomy [25]. Significant associations have been observed between increased severity of acute pain and psychological distress such as anxiety, depression, pain catastrophizing, and surgical worry [21,22]. Thus, women who elect to proceed with prophylactic mastectomy rather than breast-conserving treatment may experience more psychological distress and therefore predispose them to higher postoperative pain levels. Evidence has shown that addressing preoperative emotions and perceptions can improve outcomes including postoperative pain [26]. This provides an opportunity to potentially reduce postmastectomy pain using distinct preoperative interventions to psychologically prepare patients.

The remaining patients in the non-cancer group had gender-affirming surgery. However, we did not find a strong relationship between the transgender population and increased opioid use for our model. There is limited literature on pain in transgender patients. A recent study found that patients who underwent oncologic mastectomy were prescribed and consumed lower amounts of opioids after discharge compared to patients who underwent gender-affirming mastectomy [27]. However, another recent study found that transgender men had lower opioid consumption in the recovery room following mastectomy or breast reduction surgery than females [28]. Further research should be done to clarify and elucidate this relationship.

This data can be used to identify patients at risk for higher opioid use. Multiple studies have shown an association between acute postoperative pain and the development of chronic pain [2,13,14]. Given the current state of America’s ongoing opioid crisis, it would be beneficial to distinguish these high-risk patients and incorporate preventative measures. This model has the potential to be an important tool in precision medicine by using patients’ individual characteristics to tailor pain management strategies for high-risk patients. Intraoperatively, these patients can be treated with opioid-sparing techniques such as nonopioid alternatives, regional anesthesia, and neuraxial anesthesia. Postoperatively, measures could include involving experts in pain management with outpatient care to provide comprehensive education. Pain management resources can be more efficiently allocated by identifying these high-risk patients who could benefit the most from this resource. Furthermore, we could consider more novel regional anesthesia modalities in higher-risk patients, including ultrasound-guided intercostal cryoablation or two-level paravertebral catheters to provide a wider window of dermatomal coverage [16,17]. Additional studies should be performed to identify the intraoperative and postoperative techniques that most effectively decrease opioid use.

Despite the performance of our model, this study has several limitations. As a retrospective study, we could not collect data on other important variables such as baseline pain scores, anxiety and depression scales, or socioeconomic class. Additionally, this model was built using single-institution data and therefore should be validated to external data sets at different institutions. Future steps include performing a prospective study including these relevant variables and validating our model at other institutions.

## Conclusions

The use of opioids as part of pain management for postmastectomy patients remains a common clinical practice. Our study found factors that help predict postoperative opioid use include postmenopausal, age, race, bilateral surgery, mastectomy with tissue expander placement, cancer diagnosis, depression, substance abuse history, smoking, hypertension, and asthma. A predictive model for opioid use in patients who undergo mastectomies can identify individuals who are at higher risk of opioid use and therefore allow providers to employ targeted interventions for these patients. Additional research is necessary to determine an appropriate methodology to apply this model in clinical settings and determine the most effective preventative measures to reduce opioid use among high-risk patients.
